# DNA repair genes play a variety of roles in the development of fish embryos

**DOI:** 10.3389/fcell.2023.1119229

**Published:** 2023-03-01

**Authors:** Abhipsha Dey, Martin Flajšhans, Martin Pšenička, Ievgeniia Gazo

**Affiliations:** Faculty of Fisheries and Protection of Waters, South Bohemian Research Center of Aquaculture and Biodiversity of Hydrocenoses, Research Institute of Fish Culture and Hydrobiology, University of South Bohemia in České Budějovice, Vodňany, Czechia

**Keywords:** DNA repair, organogenesis, genotoxicity, fish, embryo

## Abstract

Embryogenesis is one of the most important life stages because it determines an organism’s healthy growth. However, embryos of externally fertilizing species, such as most fish, are directly exposed to the environment during development and may be threatened by DNA damaging factors (pollutants, UV, reactive oxygen species). To counteract the negative effects of DNA fragmentation, fish embryos evolved complex damage response pathways. DNA repair pathways have been extensively studied in some fish species, such as zebrafish (*Danio rerio*). Our literature review, on the other hand, revealed a paucity of knowledge about DNA damage response and repair in non-model aquaculture fish species. Further, several pieces of evidence underlie the additional role of DNA repair genes and proteins in organogenesis, spatiotemporal localization in different tissue, and its indispensability for normal embryo development. In this review, we will summarize features of different DNA repair pathways in course of fish embryo development. We describe how the expression of DNA repair genes and proteins is regulated during development, their organogenetic roles, and how the expression of DNA repair genes changes in response to genotoxic stress. This will aid in addressing the link between genotoxic stress and embryo phenotype. Furthermore, available data indicate that embryos can repair damaged DNA, but the effects of early-life stress may manifest later in life as behavioral changes, neoplasia, or neurodegeneration. Overall, we conclude that more research on DNA repair in fish embryos is needed.

## 1 Introduction

Genome stability is critical for the survival of a cell and, by extension, the organism. Cellular DNA is constantly under attack from a variety of lesions caused by either exogenous agents such as genotoxic chemicals or endogenous factors such as reactive oxygen species produced during normal cellular metabolism (Chatterjee and Walker, 2017). Different types of damage can result in DNA single-strand and double-strand breaks, base modification, cross-links, *etc.* As a result of the damage, cells have developed DNA repair mechanisms that can prevent the accumulation of DNA injuries. To ensure error-free replication in the adult somatic cell cycle, DNA damage is detected and repaired before or during genome replication (Hu et al., 2015).

Fish live in a variety of marine and freshwater ecosystems and are exposed to DNA-damaging factors at all stages of development. Externally fertilized fish embryos develop in water bodies, making them susceptible to DNA damage caused by xenobiotic agents like genotoxic chemicals, metals, pesticides, synthetic hormones, *etc.*, and environmental factors like radiation, heat, *etc.* Vertebrates developed several pathways of xenobiotic resistance, such as ABC transporters or UDP-glucouronyl transferases (Ugts) (Li and Wu, 2007). Ugts are a family of phase-II drug metabolizing enzymes playing role in detoxification of xenobiotics (Huang and Wu, 2010; Wang et al., 2014). Nevertheless, numerous human-made chemicals find their way inside the cells and can induce significant damage to DNA. The early developmental stages are particularly susceptible to xenobiotic influence since a development-dependent expression of *ugt* transcripts was recorded (Christen and Fent, 2014). Furthermore, genotoxicants can have long-term effects as demonstrated for *Gasterosteus aculeatus* (Santos et al., 2013) and induce epigenetic changes with transgenerational effects in fish (reviewed by Terrazas-Salgado et al., 2022). DNA replication and cell proliferation are rapid during early embryonic development, and the cell cycle progresses without G1 and G2 before the mid-blastula transition (Kermi et al., 2017). Thus, to prevent mutations in actively proliferating cells during early embryo development, DNA repair is critical (Levine, 2004; Nordman and Orr-Weaver, 2012).

**FIGURE 1:**
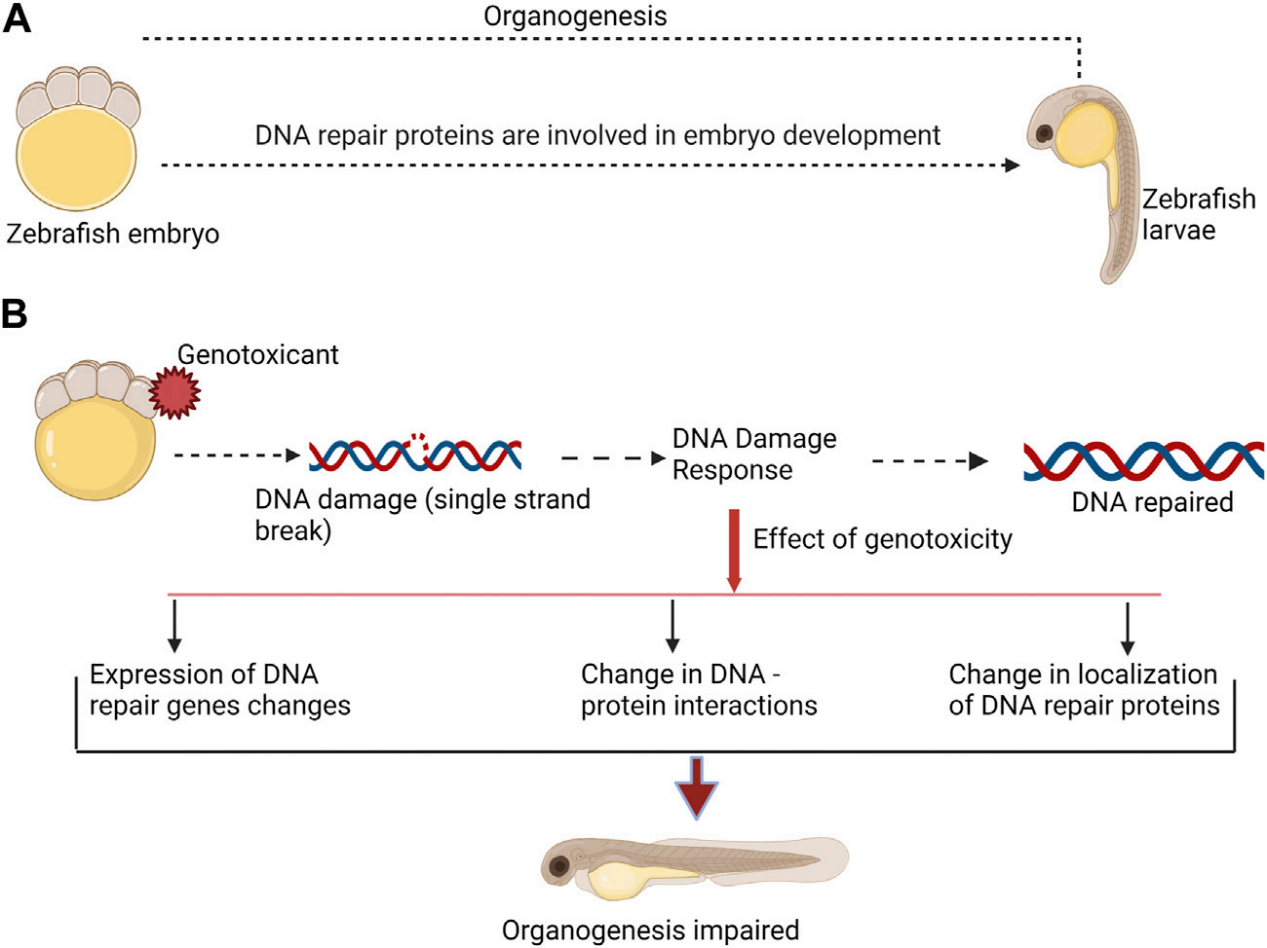
DNA repair proteins are involved in both, normal embryo development, and DNA damage response. **(A)**. During normal embryo development DNA repair proteins participate in organogenesis. **(B)**. Genotoxicant exposure at embryonic stage can impair normal organogenesis. Genotoxicants induce DNA damage, which requires activity of DNA repair proteins. DNA damage response include changes in expression of DNA repair genes and proteins, changes in DNA-protein interactions, and affect the localization of DNA repair proteins. Taken together, these alterations may affect function of DNA repair proteins in organogenesis. This can consequently result in malformation.

The majority of studies on DNA repair pathways have been conducted in zebrafish (*Danio rerio*) (reviewed by Pei and Strauss, 2013; Canedo and Rocha, 2021). Several literatures, however, described DNA repair in other fish species, including *Xiphophorus* (reviewed by David et al., 2004), *Kryptolebias marmoratus* (Rhee et al., 2011), and medaka, *Oryzias latipes* (Armstrong et al., 2002; Barjhoux et al., 2014). Base excision repair (BER), nucleotide excision repair (NER), photoenzymatic repair, direct reversal (DR), mismatch repair (MMR), homologous recombination (HR), and non-homologous end joining (NHEJ) are all proven DNA repair pathways in fish (Pei and Strauss, 2013). However, the proteins involved in DNA repair pathways appear to have more functions than just correcting DNA errors. They also take part in crucial DNA metabolism pathways during development (Shin et al., 2021). The fact that DNA damage response (DDR) proteins exhibit complex stage- and tissue-specific patterns of expression and activity (Sun et al., 2019) may indicate a role in development and organogenesis regulation (reviewed by Friedberg and Meira, 2000; Figure 1).

Furthermore, we speculate that some exogenous damaging agents can impair DNA repair protein function. Any change in the expression or localization of these proteins as a result of genotoxic stress can impact their developmental role and steer malformation during development (Figure 1). The goal of this review is to summarize the existing literature on the roles of DNA repair genes and proteins in fish embryo development and to investigate the relationship between phenotype formation and disruption of developmental pathways under genotoxic stress.

## 2 Embryogenesis in fish and DNA repair at the early embryonic stage

The DDR is a signal transduction pathway that detects damage and triggers a coordinated cellular response to protect the cell. The response to DNA damage can differ depending on the developmental stage. As a result, determining the timing of gene expression in embryos is critical for understanding the dynamics of gene regulation (Chechik and Koller, 2009).

**FIGURE 2:**
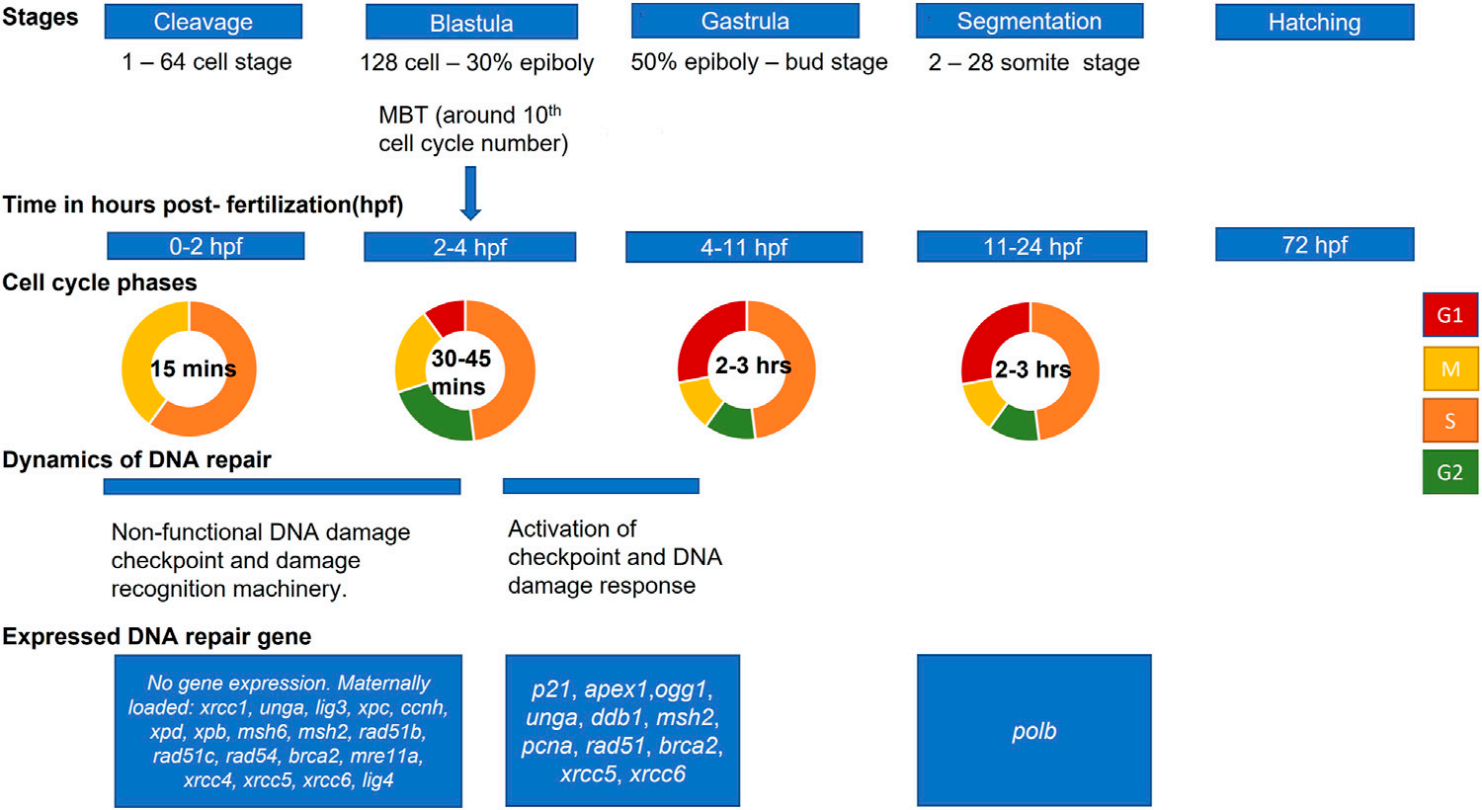
Scheme of zebrafish embryo development and main events in activation of DNA damage response. Developmental stages and approximate hours post-fertilization (at 28.5°C) for each stage are shown from cleavage (first cell divisions) to hatching (based on Kimmel et al., 1995). The mid-blastula transition (MBT) marks changes in cell cycle and activation of checkpoints. Activation of zygotic transcription at this stage is associated with expression of some DNA repair genes.

We have summarized different stages of embryo development with respect to DDR pathways (Figure 2). Most of the studies available in literature have been performed on zebrafish model; however, it is important to keep in mind that timing of development vary significantly between different fish species. Furthermore, information is scarce about DDR gene expression at different stages of development in other fish. Therefore, in the current review we present data about embryo development and development of DNA repair mechanisms mostly for zebrafish (Figure 2). Future studies should complete the gap in knowledge about DDR in embryos of non-model fish species.

Fish embryo development begins with a series of synchronous and rapid cell cycles (cleavage stage) (Kane and Kimmel, 1993). Unlike adults, embryos at the cleavage stage cycle through S and M phases without G1 and G2 (Siefert et al., 2015). Most zygotic genes are silent, and the embryo relies on maternally deposited mRNAs for development (Zhang et al., 2014). The maternally loaded DNA repair transcripts include: *xrcc1*, *unga*, *lig3* for BER; *xpc*, *ccnh*, *xpd* and *xpb* for NER; *msh6* and *msh2* for MMR; *rad51b*, *rad51c*, *rad54*, *brca2* and *mre11a* for HR; *xrcc4, xrcc5*, *xrcc6* and *lig4* for NHEJ (Honjo and Ichinohe, 2019; Silva et al., 2012; Wu et al., 2014; Figure 2).

Though some DNA repair activity is present in zygotes and early-stage embryos, their ability to recognize DNA damage and to respond is reduced. Thus, checkpoint regulations differ between embryonic and adult somatic cells (Munisha and Schimenti, 2021). DNA damage checkpoints are mechanisms that link DNA repair to cell cycle events. Checkpoints stop the cell cycle and give the damaged DNA time to get repaired, or they initiate apoptosis if repair is not possible (Hartwell and Kastan, 1994; Ford and Kastan, 2020). However, in fish embryos at cleavage stage, cell divisions are unaffected by inhibitors of DNA replication or DNA damaging agents (Ikegami et al., 1997). This fact indicates impaired checkpoint activity at early stages. Following cleavage, the mid-blastula transition (MBT) occurs, which is characterized by cell cycle lengthening, loss of cell synchrony, and activation of checkpoints (Kane and Kimmel, 1993; Ikegami et al., 1999). MBT in fish corresponds with zygotic transcription activation (Tadros and Lipshitz, 2009). The S-phase lengthens at MBT which corresponds to the need for a DNA replication checkpoint in zebrafish embryos (Siefert et al., 2015).

Additionally, machinery for detecting damaged DNA is inefficient until 4hpf in zebrafish (Honjo and Ichinohe, 2019). The 2hpf embryonic cells were found to be incapable of forming phosphorylated H2AX foci (γH2AX). The formation of γH2AX is a conserved mechanism of early recognition and response to DNA double-strand breaks (Podhorecka et al., 2010). As a result, available data indicate that the mechanisms such as DNA damage recognition, checkpoint activation, and apoptosis are compromised at an early stage of fish embryo development. This could be an adaptation strategy to ensure embryonic cell divisions take place even in adverse conditions (Warkentin, 1995; Kermi et al., 2019). The delay in activation of checkpoint after MBT can provide a time frame for the induction of cytogenetic lesions by DNA-damaging agents in the developing embryo (Ikegami et al., 1997).

After cleavage stage, blastulation starts from 128-cell stage until the time of gastrulation (Kimmel et al., 1995; Figure 2). After MBT, yolk syncytial layer forms and epiboly starts. Further, at gastrulation stage, morphogenetic cell movements result in primary germ layers and embryonic axis. The epiblast and hypoblast are formed, which will give rise to ectoderm, mesoderm, and endoderm. At segmentation stage, somites develop and organogenesis starts. The tail bud becomes prominent, and first body movements appear. At the next stage, pharyngula, the body axis straightens from its early curvature around the yolk sac, and circulation, pigmentation, and fins begin to develop. Finally, hatching occurs when morphogenesis of primary organs is complete, cartilage developed in head and pectoral fin (Kimmel et al., 1995).

## 3 DNA repair pathways in developing fish embryos are stage-specific

The expression of DNA repair proteins is temporally regulated during organogenesis and can be localized to specific organs and tissues (Vinson and Hales, 2002). We will summarize briefly the stage-specific activity of the major DNA repair pathways in developing embryos (Figure 2; Table 1).

**TABLE 1:** Summary of the expression, localization and developmental role of zebrafish DNA repair genes and proteins during embryonic development. ND stands for “not determined”. KD stands for knockdown.

DNA repair gene (protein)	Developmental stage of expression	Localization	Knockdown-induced phenotype	References
*p21*	From 6 hpf	ND	ND	Honjo and Ichinohe, (2019)
*apex1*	At all stages; localization starts at 48hpf	Head region	KD affects red blood cells, eyes, brain, notochord, and heart	Wang et al., 2006; Pei et al., 2019
*ogg1*	At all stages from 1 cell to 8dpf	Ventricular zone of mid-brain, retina and peripheral nerve, heart	Abnormal brain	Gu et al., 2013
			Shortened ventricle and atrium of heart	Yan et al., 2013
*unga*	At all stages; enriched expression from segmental stage	Neural tube and tail bud	Embryonic lethality during segmentation	Wu et al., 2014
*ddb1*	At all stages	CNS, head skeleton, pharyngeal region, and endoderm	Abnormal eyes, brain and head skeleton, defects in jaw	Hu et al., 2015; Shin et al., 2021
*msh2*	At all stages	Head region and tissues around eyes	Higher susceptibility to tumor in eye region and abdomen	Yeh et al., 2004; Feitsma et al., 2008; Honjo and Ichinohe, 2019
*pcna*	At all stages	Brain, spinal cord, kidney, spermatogonia	Reduced head and eye size, curved body trunk formation, and increased apoptosis in caudal hematopoietic tissue	Leung et al., 2005; Shin et al., 2021
*eepd1* (EEPD1)	ND	ND	Impaired somitogenesis, developmental delay	Chun et al., 2016
*rad51*	At all stages	ND	Development of infertile male-biased individuals, possible impairment of meiosis	Thisse and Thisse, 2004; Shin et al., 2021
*brca2*	At all stages	Brain, eye, ear, kidney	Abnormal kidney development, aberration in gonad development; male-biased phenotype, lack of spermatozoa in testis, possible impairment of meiosis, infertility	Kroeger et al., 2017; Shive et al., 2010; Shin et al., 2021
*xrcc5* (Ku80)	From 2 cell to 24hpf; restricted expression starts at 24hpf	Retina, anterior CNS and otic vesicles	Abnormal brain only when exposed to irradiation stress	Bladen et al., 2005
*xrcc6* (Ku 70)	From 2cell to 24 hpf; localization onset at 24hpf	Retina and proliferative regions of brain	Abnormal brain only when exposed to irradiation stress	Bladen et al., 2007

### 3.1 Base excision repair

BER is a critical repair system that occurs at all stages of fish development (Pei and Strauss, 2013). Small base lesions caused by deamination, oxidation, or methylation are repaired by BER. The pathway consists of several steps of DNA damage recognition, replacement of the damaged base and restoration of DNA structure. In more details, DNA glycosylases recognize the mismatched or modified DNA base (Chatterjee and Walker, 2017). 8-oxoguanine DNA glycosylase (Ogg1), uracil-DNA glycosylases (Udg), and Nth-like DNA glycosylase 1 (Nthl1), and others are among these glycosylases. Following damage recognition, DNA glycosylase removes the inappropriate base, resulting in an apurinic or apyrimidinic site, which is then cleaved at the 5ʹ site by apurinic endonuclease (Ape1), leaving a free 3’ site. Subsequently, DNA polymerase β (Polβ) replaces the missing nucleotide and the resulting nick is ligated by Xrcc1 complex—Lig3α to reinstate the original DNA structure (Pei and Strauss, 2013; Canedo and Rocha, 2021).

Notably, BER activity in zebrafish eggs and early-stage embryos has several distinct characteristics and is less efficient than in adults (Fortier et al., 2009). Cell-free extracts from eggs were capable of removing U-G mispair and inserting the correct base pair. However, the efficiency of Udg and Ape1 in 3.5 hpf old zebrafish embryos is lower than in adults (Fortier et al., 2009). Moreover, the same study showed that early-stage zebrafish embryo possesses an additional Mg^2+^-dependent endonuclease (Apex) while adult possesses a single major Ape1.

Although mRNA for Polβ was present at all stages of embryogenesis, zebrafish eggs, and embryos lacked replicative Polβ protein until gastrulation; instead, a DNA polymerase sensitive to aphidicolin was present (Fortier et al., 2009). Pei et al. (2011) showed that Apex regulates the transcription of *creb1* and its binding partners, which in turn regulates Polβ expression in embryos of zebrafish before gastrulation. It is unclear whether all fish species lack Polβ protein during early development or if this is a feature unique to zebrafish. However, another study found that Polβ had very low activity in the early stages of *Misgurnus fossilis* (loach) development when compared to polymerases α and γ (Mikhailov and Gulyamov, 1983).

### 3.2 Direct reversal

DR primarily addresses alkylation at the O^6^ position of guanine (O^6^-alkylguanine) by utilizing O—methylguanine—DNA-methyltransferase (O^6^-Mgmt), which removes the alkyl group from O^6^ position of guanine in damaged DNA (Gasch et al., 2017). Rhee et al. (2011) revealed lower transcript levels of *mgmt* mRNA in embryos at 12 dpf and juveniles of *K. marmoratus* compared to adults. This fact could indicate more susceptibility to DNA damage by alkylating agents during early developmental stages.

### 3.3 Nucleotide excision repair

NER pathway mainly deals with helix-distorting lesions such as pyrimidine dimers, DNA cross-links, and bulky adducts. This pathway is thus in charge of repairing UV-induced photoproducts as well as DNA damage caused by chemical carcinogens and chemotherapeutic drugs. The modified base is first recognized by the Xpc (Xeroderma pigmentosum group C) damage recognition complex, which then causes the DNA helix to unwind. Alternatively, the UV-damaged DNA binding complex (UV-Ddb) is an auxiliary damage recognition complex composed of two subunits, Ddb1 and Xpe (Ddb2) (Dexheimer, 2013). Following the detection of the initial lesion, transcription factor IIH (TfIIh) and Xpa are recruited to the site to confirm DNA damage. The DNA is then cleaved at 3ʹ and 5ʹ positions of damage by Xpg and Xpf nucleases, removing the lesion. Finally, using an undamaged strand as a template, DNA polymerases resynthesize the gap, and DNA ligase seals the nick in the repaired strand.

Expression and activity of proteins involved in the NER pathway seem to be developmentally regulated (Hsu et al., 2001). Hsu et al. (2001) suggested that distinctive UV-damaged-DNA binding factors are expressed in zebrafish embryos at different developmental stages in contrast to adults. The same study revealed the development-regulated expression of Xpa in zebrafish. The Xpa protein expression was not detected until 84 hpf. Later, Shen et al. (2017) confirmed that the vitellogenin-1-like protein in zebrafish embryos plays a role of UV-damaged-DNA binding factor and revealed its function in post-incision step of NER (Shen et al., 2017).

### 3.4 Mismatch repair

MMR is an evolutionarily conserved post-replicative repair pathway for the repair of mismatched bases, insertion, and deletion of nucleotides. MMR begins with the recognition of base mismatches by MutSα heterodimer (Msh2/Msh6) and MutSβ heterodimer (Msh2/Msh3) (Chatterjee and Walker, 2017). Following that, molecules like proliferating cell nuclear antigen (Pcna), replication factor C, MutLα, and exonuclease one are recruited to the complex, resulting in mismatch dissociation (Liu et al., 2017; Pećina-Šlaus et al., 2020). The excision gap is stabilized by replication protein A (Rpa) (Genschel and Modrich, 2003). Finally, DNA polymerase δ bridges the gap and DNA ligase I connect the filament (Li, 2008; Li, 2013).

*Msh2* and *msh6* transcript levels were higher in 12–36 hpf than in 84 hpf zebrafish larvae (Yeh et al., 2004). Furthermore, *msh2* mRNA production gradually decreased in 60–120 hpf zebrafish. The authors linked *msh2* gene expression downregulation to a decrease in the number of actively growing cells as zebrafish matured. It is unclear, however, how differentiation or cell growth activates the Msh expression in fish embryos.

### 3.5 Homologous recombination

HR is a template-directed, high-fidelity repair pathway that is used to repair double strand breaks (DSBs), DNA gaps, and DNA interstrand cross-links. At first, MRN (Mre-Rad50-Nbs1) complex initiates repair by recognizing and binding DSB (Chatterjee and Walker, 2017). The protein exonuclease/endonuclease/phosphatase domain-1 (Eepd1) is essential for initiating HR and restarting replication of stalled forks (Wu et al., 2015). Rpa then binds to the single-stranded DNA tails, activating the Atr kinase pathway. This causes Rad51 recombinase to invade the homologous region and replace Rpa. Finally, DNA polymerase δ extends and seals the invading chain with DNA ligase (Reinardy et al., 2013).

Brca1 and Brca2 are the two main factors playing a critical role in HR pathway by recruiting Rad51 to the vicinity of DSBs (Vierstraete et al., 2017). Vierstraete et al. (2017) observed active participation of Brca1 and Brca2 in HR pathway of 72 hpf zebrafish embryo suggesting the involvement of HR in DSB repair in developing embryos of zebrafish. Further, CRISPR-Cas9-mediated knockdown of HR genes, such as *mre11a* and *wrn*, led to mortality at 60 dpf (Shin et al., 2021).

### 3.6 Non-homologous end joining

NHEJ is a major pathway for repairing DNA DSBs that is active during G1 and early S phases. The NHEJ pathway is primarily mediated by five genes: *xrcc5,* which encodes the Ku80 protein, *xrcc6* which encodes Ku70 protein, *lig4* and *xrcc4,* which encode two subunits of DNA ligase, *prkdc* which encodes DNA-dependent protein kinase (Bladen et al., 2005; Bladen et al., 2007). The Ku70/80 heterodimer binds to two ends of the broken DNA molecule, forming a protein-DNA complex that attracts the DNA-dependent protein kinase catalytic subunit. Following this, the ends of broken DNA molecules are processed using different enzymes like Artemis, polymerase λ and µ, tyrosyl-DNA phosphodiesterase, or polynucleotide kinase. Finally, DNA is sealed by ligase 4-Xrcc4 complex (Sonoda et al., 2006; Canedo and Rocha, 2021).

It has been shown that knockdown of *lig4* (ligase four gene) in zebrafish embryos resulted in significant impairment of NHEJ pathway and mortality at 24 hpf (Liu et al., 2012). In contrast, the same study showed that morpholino-induced knockdown of *rad51* impaired HR pathway and led only to malformations in 48 hpf embryos. It can be suggested that NHEJ is the predominant DSB repair pathway during early embryo development (Liu et al., 2012; Vierstraete et al., 2017; Canedo and Rocha, 2021). Whereas HR is active at later stages and plays important role in organogenesis (see below).

## 4 Developmental role of DNA repair genes and proteins

Apart from their function in DNA repair pathways some of the above-mentioned genes and proteins play important roles in fish embryo organogenesis. In this part we will briefly summarize what is known about dual functioning of DNA repair factors. Most of the studies available in literature evaluated the role of DNA repair in organogenesis through knockout and knockdown of corresponding genes. This allows researchers to evaluate a resulting embryo phenotype, indicating which developmental pathway requires a specific DNA repair protein. Another approach focuses on localization of gene or protein expression inside the embryo. We have summarized available information on several DNA repair genes concerning their localization and knock-down specific phenotypes in Table 1.

### 4.1 BER pathway

Several proteins from the BER pathway have been found to be involved in the development of fish embryos. Wang et al. (2006), for example, demonstrated that knocking down *apex1* in early zebrafish allowed the embryo to survive up to seven dpf with pericardial edema and a lack of circulating red blood cells. This study demonstrated that *apex1* plays a role not only in cardiovascular and hematopoietic development but also in neural and notochord development (Wang et al., 2006). Further, Pei et al. (2019) demonstrated that loss of Apex1 protein in zebrafish embryos resulted in abnormal distribution and loss of four key brain transcription factors (*fezf2*, *otx2*, *egr2a*, and *pax2a*). That led to abnormal brain development including a small head and distortions in the ventricle (Pei et al., 2019).

Wu et al. (2014) used whole mount *in situ* hybridization to detect the spatiotemporal expression of maternally supplied *unga* (uracil DNA glycosylase a) mRNA in zebrafish embryos and larvae. During the early cleavage stage, the *unga* was uniformly distributed, but it localized to neural tube and tailbud during segmentation at 5hpf. Its knockdown resulted in an increase in global DNA methylation, a decrease in overall transcriptional activity in the nucleus, and embryonic lethality during the segmentation period (Wu et al., 2014).

Several studies investigated the role of Ogg1 in zebrafish embryo development. Gu et al. (2013) studied spatiotemporal expression of *ogg1* in the zebrafish embryo. They provided evidence of localization of *ogg1* mRNA in the ventricular zone of mid-brain in zebrafish at 17–24 hpf, in retina (at 24–36 hpf), and in peripheral nerve (at 48 hpf). Further, loss of *ogg1* resulted in the development of abnormal brain vesicles with diminished ventricle size and destructed mid-brain/hind brain boundary (Gu et al., 2013). Apart from its function in brain development, Ogg1 seems to play role in heart and circulatory system development. Yan et al. (2013) explored the significant role of *ogg1* in cardiomyocyte formation in zebrafish. Knock-down of *ogg1* resulted in marked reduction in expression of *nkx2.5* (factor which specify the fate of cardiac cell during gastrulation) and repression of *foxh1* (an important partner of *ogg1* in cardiac development in response to DNA damage) (Yan et al., 2013). Loss of *nkx2.5* and *foxh1* led to increased apoptosis, decreased proliferation, and embryonic lethality at five dpf (Yan et al., 2013).

### 4.2 NER pathway

Hu et al. (2015) investigated the role of *ddb1,* an important component of the NER pathway, in the development of zebrafish embryos. The *ddb1* ^*m863*^ mutant allele was created through chemical N-ethyl-N-nitrosourea mutagenesis. Ddb1 protein was truncated because of the mutation. Obtained mutants showed a prominent phenotype with reduced size and abnormal structure of the eyes, brain, and head skeleton on the three dpf. They also reported ubiquitous expression of *ddb1* mRNA in early wild-type embryos, which got restricted to the central nervous system (CNS), head skeleton, pharyngeal region, and endoderm during the third day of development. A correlation was also discovered between *pcna,* a proliferation marker, and *ddb1* expression levels, indicating that cell proliferation is dependent on high levels of *ddb1* expression (Hu et al., 2015). Similarly, Shin et al*.* (2021) created homozygous mutant for ddb1 knockout using CRISPR/Cas9-mediated mutagenesis and demonstrated that ddb1−/− mutants displayed anterior malformation with defects in the eye and jaw structure as early as six dpf when compared to non-mutant zebrafish embryos. Though the phenotypes observed in both studies were similar, the timing of phenotype formation differed. This may be due to genetic compensation in CRISPR mutants (Shin et al., 2021).

### 4.3 MMR pathway

Several studies investigated the role of mismatch repair proteins, Msh2, Msh6 and Pcna, in zebrafish embryo development. Thus, *msh2* expression was found in the brain and around the eye primordium of zebrafish embryos at the six-somite stage (12 hpf) (Yeh et al., 2004). In pharyngula stage (24 hpf), *msh2* mRNA synthesis was localized in head region. At 48 hpf and 60 hpf, expression of *msh2* became concentrated in the telencephalon, tissues around the eyes, and the fourth ventricle (Yeh et al., 2004).

Furthermore, it has been shown that *msh2* and *msh6* knockout zebrafish mutants are prone to tumor development and primarily develop neurofibromas/malignant peripheral nerve sheath tumors in the eye region and abdomen (Feitsma et al., 2008). Interestingly, location of tumors corresponded to mRNA expression at embryonic stage. Also, levels of *msh2* and *msh6* were higher in actively differentiating cells (Yeh et al., 2004). It was documented that Msh2 protein is involved in proliferation, cell cycle progression, and apoptosis in mammalian tissues and tumors (reviewed by Seifert and Reichrath, 2006). However, it is not clear how exactly Msh2 protein expression is associated with cell cycle regulation, particularly at embryonic stage in fish.

Probably the most known example of DNA repair protein playing an important role in development is Pcna. This protein is involved in both, proliferation, and DNA repair. In zebrafish embryos, Shin et al. (2021) found that a frameshift mutation in pcna caused reduced head and eye size, curved body trunk formation, and increased apoptosis in caudal hematopoietic tissue (Shin et al., 2021). Leung et al. (2005) previously demonstrated that Pcna is expressed in the brain, spinal cord, and intermediate cell mass of zebrafish embryos at 24 hpf (Leung et al., 2005). Thus, the malformations observed in pcna mutants by (Shin et al., 2021) correlate with tissues expressing the highest levels of Pcna during development.

### 4.4 DSB repair pathways

The two main pathways of DSB repair seems to play an important role in fish embryo development. For example, Eepd1 promotes HR and inhibits NHEJ. Chun et al. (2016) proposed that Eepd1 endonuclease is involved in somitogenesis and maintains genome stability in zebrafish embryos during endogenous replication stress caused by rapid cell division during embryogenesis. Eepd1 protein knockdown caused developmental delays, increased lethality, and malformations. Though NHEJ is a predominant pathway for DNA repair during the early stages of embryo development, HR is also involved in genome stability maintenance during cell proliferation (Chun et al., 2016).

Shin et al. (2021) generated zebrafish mutants of genes related to HR repair *via* multiplexed CRISPR mutagenesis. Interestingly, they observed that homozygous mutants for *brca2, blm, rad54l,* and *rad51* developed male-biased phenotype. Mutants of *rad54l* showed normal fertility while in mutants of *brca2* and *rad51* primordial germ cells failed to survive and proliferate*,* leading to infertile males (Shin et al., 2021). Shive et al. (2010) reported indispensable role of *brca2* in ovary development during sexual differentiation in zebrafish line with a mutation in *brca2* exon 11 (*brca2* ^Q658x^). Homozygous mutants *brca2* ^Q658x^ developed as infertile males with meiotic arrest at spermatocytes. This revealed *brca2* is not only required for ovarian development but also for spermatogenesis (Shive et al., 2010). The DSB repair proteins, particularly Brca2, Dmc1, and Rad51, play an important role in meiosis in zebrafish (reviewed by Sansam and Pezza, 2015; Imai et al., 2021). Though sex differentiation occurs in zebrafish at 20–25 dpf, disruption of HR proteins at early stages of development could lead to abnormal gamete development and apoptosis. Moreover*,* impairment of HR pathway was associated with an increased risk of tumorigenesis in gonad (Shive et al., 2010; Shin et al., 2021).

Several NHEJ pathway factors have been found in the developing brain and CNS of zebrafish. Thus, Ku80 mRNA is uniformly expressed in 2-cell blastomeres through gastrulation, until the end of somitogenesis in 24 hpf zebrafish embryos and becomes spatially restricted in developing retina, anterior CNS, and otic vesicle (Bladen et al., 2005). Similarly, Ku70 mRNA expression increases at 24 hpf embryos, remains elevated until 72 hpf, and is spatially expressed in developing CNS, including ventricular zones of the brain and presumptive ganglion cell layer of the retina (Bladen et al., 2007). However, only when the embryos were exposed to low doses of ionizing radiation did morpholino-mediated knockdown of Ku70 affect zebrafish embryogenesis (Bladen et al., 2007).

The studies mentioned above show that DNA repair genes are expressed spatiotemporally during organogenesis. However, their direct role in organogenesis and the mechanism underlying DNA repair proteins’ multifunctionality have yet to be studied. More research into the developmental role of DNA repair proteins is needed to understand the etiology of defects during organogenesis in response to genotoxicants.

## 5 Xenobiotics effects on DNA repair pathways

Genotoxicants are substances found in the environment that directly bind to DNA or affect the DNA repair enzymes causing damage to DNA. Cells detect and respond to genotoxic stress by altering gene transcription (Gasch et al., 2017), protein abundance (Nouspikel, 2009; Soufi et al., 2009), protein post-translational modification (Peng et al., 2003; Ptacek et al., 2005), or protein intracellular localization (Tkach et al., 2012). Pollutants can also cause DNA damage by interfering with DNA repair mechanisms. We hypothesize that any disruption in the expression of DNA repair genes in response to genotoxicants during embryonic development can perturb their developmental role, causing organogenesis to be disrupted. Table 2 summarizes the effect of different xenobiotics on developing fish embryos, DDR gene expression, and induced phenotypes. In this chapter we review studies on toxicant exposures associated with changes in DDR gene expression, and phenotype formation.

**TABLE 2:** Effect of xenobiotics on expression of DNA repair genes and proteins during fish embryogenesis and corresponding abnormality induced.

Fish species	Xenobiotic	Impacted DNA repair genes and proteins	Induced phenotypic abnormality	Time point at which abnormality was recorded	References
Zebrafish	Dibutyl phthalate	*ogg1*, *lig3*, *unga*, *pcna*, *pold*, *fen1*, and *lig1*	Reduced hatching rate, body length, and increased deformity	72 hpf	Lu et al. (2021)
			Inhibition of swimming activity	96 hpf	
Zebrafish	Mono-(2-ethylhexyl) phthalate	*ogg1*, *nthl1*, *apex1*, *parp1*, *lig3*, *pcna*, and *polb*	Reduced hatching rate, body length, and increased deformity	72 hpf	Lu et al. (2021)
			Inhibition of swimming activity	96 hpf	
Zebrafish	Di-(2-ethylhexyl) phthalate	*ogg1*, *parp1*, *pcna*, *fen1*, *pold*, and *lig1*	Not detected		Lu et al. (2021)
Zebrafish	Graphane oxide	*apex1, ogg1, polb,* and *creb1*	Yolk sac edema, tail flexure and spinal curvature	120 hpf	Lu et al. (2017)
					Ren et al. (2016)
Medaka	Methylpyrene	*ogg1*	Spinal deformity, yolk sac desorption and craniofacial abnormalities	4 dpf	Barjhoux et al. (2014)
			Cardiovascular injuries	6–7 dpf	
Zebrafish	Cypermethrin	*ogg1*	Body axis curvature	24 hpf	Shi et al. (2011)
			Pericardial edema and enlarged yolk sac	96 hpf	
Zebrafish	Cadmium	*xpc,* UV-*ddb2, msh2, msh6,* and *ogg1*	Apoptosis in brain	16 dpf	Ling et al. (2017)
					Hsu et al. (2013)
					Monaco et al. (2017)
Zebrafish	Mercury	*msh2*, *msh6*, Msh6	Not detected	Not dectected	Ho et al. (2015)
Zebrafish	EE2	*Xpa*	Not detected	Not detected	Notch and Mayer (2013)
Sterlet	Benzo [a]pyrene	*Xpc*	Not detected	Not detected	Gazo et al. (2021)
Sterlet	CPT	*xpc*, *rad50*, *xpa*, *xrcc1*, *msh2*, *rpa1*, *ercc5*, *pold3*, *ercc2*, *fen1*, *blm*, *rad51ap1*, *nbn*, and *eme1*	Skeletal malformation, delayed development	8 dpf	Gazo et al. (2021)
Zebrafish	Fenthion	*rad51, rad18, xrcc2,* and *xrcc6*	Reduced hatching	72 hpf	Wahyuni et al. (2021)

In italic are names of genes impacted by a xenobiotic.

Phthalate acid esters are widely used plasticizers frequently detected in water that have been reported to cause oxidative DNA damage (Berge et al., 2013). Exposure of four hpf zebrafish embryo for 96 h to phthalate esters, including dibutyl phthalate (DBP), mono-(2-ethylhexyl) phthalate (MEHP), and di-(2-ethylhexyl) phthalate (DEHP), resulted in altered expression of BER pathway (Lu et al., 2021). The mRNA levels of *ogg1*, *lig3*, *unga*, *pcna*, *pold*, *fen1*, and *lig1* were increased after DBP exposure. Exposure to DEHP led to upregulated mRNA levels of *ogg1*, *parp1*, *pcna*, *fen1*, and *lig1*, and downregulated *pold* expression. The mRNA levels of *ogg1*, nthl1, apex1, parp1, lig3, pcna, and polb increased after 10 and 25 µM MEHP exposure while decreasing at 50 µM (Lu et al., 2021). Interestingly, the changes in gene expression were associated with changes in behavior, malformation, and hatching rates in all treatments.

Graphene oxide (GO) is a carbon nanomaterial with numerous applications in biology and medicine (Ni et al., 2013). GO exposure has been linked to oxidative DNA damage (Seabra et al., 2014). After exposing blastula-staged zebrafish embryos to GO nanoparticles for 24 h, the genes *apex1, ogg1, polb,* and *creb1* were upregulated (Lu et al., 2017). The authors found no significant differences in embryo morphology, survival or hatching rate. Treatment with the same nanoparticles at 72 hpf, on the other hand, was linked to larval malformations, and neurotoxicity (Ren et al., 2016).

When exposed to sediment-spiked methylpyrene from one dpf to nine dpf, medaka (*O. latipes*) showed an increase in *ogg1* expression (Barjhoux et al., 2014). Methylpyrene is a polycyclic aromatic hydrocarbon (PAH), a potential carcinogen, released into the environment by incomplete combustion of organic matter, fossil fuels, and other sources. In this study, there was no significant increase in the level of DNA strand breaks after treatment, which could be due to an efficient DNA damage repair mechanism (Barjhoux et al., 2014). Non-etheless, *ogg1* upregulation was linked to cardiovascular injuries and, to a lesser extent, skeletal deformities in medaka embryos. This is consistent with Yan et al. (2013), who proposed that *ogg1* plays a role in cardiomyocyte formation.

Pesticides are commonly used in agriculture, but they have been linked to oxidative stress in non-target organisms (Yang et al., 2020). Thus, fenvalerate, a pyrethroid insecticide that causes oxidative DNA damage, has been shown to reduce *ogg1* gene expression in 24–96 hpf zebrafish embryos (Gu et al., 2010). Similarly, Shi et al. (2011) discovered that when four hpf zebrafish embryos were exposed to cypermethrin (an insecticide used in agriculture; that causes oxidative DNA damage) until 96 hpf, the expression of *ogg1* was downregulated and the expression of *tp53* was upregulated. Downregulation of *ogg1* expression was associated with skeletal malformations and increased apoptosis in the brain region in both studies, which is similar to the *ogg1* knockdown phenotype observed by Gu et al. (2013) (Table 2). Overall, these studies showed that when fish embryos were exposed to xenobiotics which changed gene expression in BER pathway, these exposures were associated with skeletal malformations, neurotoxicity and cardiovascular injuries.

Several studies reported alterations in DDR gene expression in response to heavy metals and xenobiotics. Ling et al. (2017) detected downregulation of *xpc*, and upregulation of UV-*ddb2* and *ogg1* genes when one hpf zebrafish embryos were exposed to Cd^2+^ and paraquat for 9 h. The authors suggested that the observed upregulation of NER gene, *ddb2*, is associated with oxidative stress. Cadmium-induced oxidative stress also ensued downregulation in the expression of MMR genes, *msh2* and *msh6*, in one hpf zebrafish embryos when subjected to sub-lethal concentrations (3–5 µM) of Cd^2+^ (Hsu et al., 2013). The inhibition of Msh6 protein binding activity was recorded after exposure of one hpf zebrafish embryos to Cd^2+^ (Ho et al., 2015). Exposure to Cd^2+^ during zebrafish embryo development was not associated with significant mortality or teratogenicity at larval stage but led to changes in behavior compared to control (Shankar et al., 2021). Furthermore, Monaco et al. (2017) reported an increased rate of apoptotic events in the brain of embryos after 24 h of treatment with Cd^2+^ (Monaco et al., 2017). This is in agreement with the localization of *ogg1, ddb1,* and *msh2* expression (Table 1). However, further studies are needed to show the possible long-term consequences of cadmium-induced neurodegeneration in fish.

Hg^2+^ has been shown to cause DNA strand breaks through oxidative stress and to impede DNA repair machinery (Rooney, 2007; Barcelos et al., 2011). Hg^2+^ had different effects on Msh6 protein synthesis and *msh2*, *msh6* mRNA expression (Ho et al., 2015). Msh6 protein synthesis was inhibited without causing *msh6* mRNA expression to be downregulated. It was proposed that Hg^2+^ could target a DNA repair protein function rather than transcription. Chang et al. (2017) then demonstrated that Hg^2+^ inhibited NER-associated damage incision activity in zebrafish embryos. Interestingly, Hg^2+^ exposure did not inhibit NER factor gene expression but did affect endonuclease activity, such as that of Xpg.

17α-ethinylestradiol (EE2) is a semi-synthetic hormone used in oral contraceptives and is known to promote mutation (Notch et al., 2007; Notch and Mayer, 2013). Co-exposure of 17α-ethinylestradiol (EE2) and its metabolite, estrone (E1), elicited an increased NER gene transcription, namely, *xpa* at 48 hpf and 72 hpf zebrafish embryos (Notch and Mayer, 2013). However, the ability of EE2 to cause DNA damage has not been described.

Benzo [a]pyrene (BaP) is a carcinogen and a model PAH produced by incomplete combustion of organic material (Soltani et al., 2019). DSBs and increased expression of *xpc* were observed in sturgeon (*Acipenser ruthenus*) embryos exposed to BaP from two cell stage to eight dpf (Gazo et al., 2021). Changes in *xpc* expression were associated with higher mortality when compared to controls. In contrast to zebrafish (Šrut et al., 2015), sterlet embryos effectively repaired DNA damage and did not show malformations by eight dpf (Gazo et al., 2021). This finding could point to the species-specificity of DDR mechanisms.

Camptothecin (CPT) is an inhibitor of DNA topoisomerase I widely used as an antitumor drug. CPT had an effect on the transcriptome profile of sturgeon embryos, according to Gazo et al. (2022). CPT treatment increased the expression of *xpc*, *rad50*, *xpa*, *xrcc1*, *msh2*, *rpa1*, *ercc5*, *pold3*, *ercc2*, *fen1*, *blm*, *rad51ap1*, *nbn*, and *eme1*. Upregulation of these genes was linked to a higher rate of malformations and decreased embryo viability. Embryos exposed to CPT during neurulation, on the other hand, showed only a slight increase in the expression of genes involved in DNA repair. Because viability and hatching rates in this group were comparable to those in the control group, these findings highlight stage-dependent sensitivity to CPT exposure in sturgeon embryos.

Fenthion is an organophosphate pesticide that causes oxidative stress and DNA damage, resulting in a decrease in the expression of the HR genes *rad51* and *rad18* in zebrafish embryos exposed for 24 h (Wahyuni et al., 2021). Further, 48 h of exposure inhibited the expression of *rad51, rad18, xrcc2,* and NHEJ genes (*xrcc6/*Ku70). In addition, exposure to terbufos (another organophosphate pesticide) for 48 h inhibited the expression of *rad51, rad18, xrcc2, xrcc6/*Ku70 (Wahyuni et al., 2021). In the same study, combined fenthion and terbufos exposure for 48 h increased the expression of *rad51* and *xrcc2*. The authors hypothesized that single pesticide exposure disrupted DSB repair pathways, whereas terbufos and fenthion combined showed less genotoxicity. Future studies should investigate whether xenobiotic-induced changes in HR gene expression at embryo stage could lead to impaired meiosis, fertility, or sex differentiation at later stages of development.

So far, numerous studies considered DNA damage as an endpoint of xenobiotic toxicity (reviewed by Kienzler et al., 2013; Canedo and Rocha, 2021). However, several toxicants can decrease the expression of key genes involved in DDR, subsequently causing the cell defenseless to DNA damage (Notch and Mayer, 2013). This necessitates future studies on the vulnerability of DNA repair pathways to stress. In this review, we discussed the various roles of DNA repair genes and proteins in fish embryogenesis. We also summarized studies in which a change in DNA repair genes was linked to malformation during development. However, few studies have looked into the relationship between the type of DNA damage, changes in DDR gene expression, and embryo phenotype (Gu et al., 2010; Shi et al., 2011; Lu et al., 2017). Thus, more research is needed to understand the relationship between DDR and phenotype formation, specifically, how changes in expression level, protein post-translational modifications, and protein translocation under genotoxic stress may affect embryo development.

## 6 Conclusion and perspectives

The rapidly developing embryos of externally fertilizing fish are exposed to environment or hatchery conditions where they deal with numerous DNA damaging factors. Inhibition of apoptosis and checkpoints during early embryo development indicates sensitivity to genotoxic damage. Cells proliferating with damaged DNA can acquire mutations, slowing the growth of the individual fish and, in the long run, the entire population. Thus, efficient DNA repair machinery is critical for avoiding damage tolerance.

Lethality can result from defects in the expression of DNA repair genes. Proteins involved in DDR are also vulnerable to damage. As a result, it is critical to identify the complex pathways by which DDR proteins participate in organogenesis. Overall, available literature indicates that alterations in BER pathway gene expression could affect cardiovascular system, CNS, brain development, and lead to changes in behavior (Wang et al., 2006; Gu et al., 2010; Gu et al., 2013; Yu et al., 2013; Barjhoux et al., 2014; Pei et al., 2019; Lu et al., 2021). Similarly, Cd^2+^-induced stress led to alterations in NER pathway and downregulation of MMR genes, which could be related to observed neurodegeneration (Hsu et al., 2013; Ling et al., 2017; Monaco et al., 2017; Shankar et al., 2021). However, more studies are required to understand changes in expression, structural modification, translocation, and inhibition undergone by repair proteins in response to genotoxicants. These future studies are necessary to understand the link between genotoxicant exposure and resulting phenotype formation.

Furthermore, the efficacy of DNA repair mechanisms appears to be stage dependent. As a result, it is necessary to describe the DDR pathways involved at various stages of fish embryo development to determine which stages are most vulnerable to stress and require immediate attention. Aside from zebrafish, there is a striking lack of research on DDR in other fish species; however, damage sensitivity and DDR may be species-specific. As a result, we believe that more research is needed to elucidate DDR in non-model species important for aquaculture, as well as more research to understand the level of DNA damage tolerance and repair in non-model fish species at the embryonic stage. This could lead to the discovery of new DDR genes and proteins, particularly during the embryonic stage.

## Glossary

BER: Base excision repair
NER: Nucleotide excision repair
DR: Direct reversal
MMR: Mismatch repair
HR: Homologous recombination
NHEJ: Non-homologous end joining
DDR: DNA damage response
MBT: Mid-blastula transition
OGG1: 8-oxoguanine DNA glycosylase
Unga: Uracil-DNA glycosylases
NTHL1: Nth-like DNA glycosylase one
APE1: Apurinic endonuclease
Polβ: DNA polymerase β
XRCC1: X-ray cross-complementing protein one
Lig3α: DNA ligase 3α
O^6^-MGMT: O—methylguanine—DNA-methyltransferase
XPC: Xeroderma pigmentosum group C
UV-DDB: UV-damaged DNA binding complex
XPE: Xeroderma pigmentosum complementation group E protein
XPA: DNA repair protein complementing XP-A cells
MSH: MutS homolog
PCNA: Proliferating cell nuclear antigen
RPA: Replication protein A
DSB: Double strand break
EEPD1: Exonuclease/endonuclease/phosphatase domain-1
ATR: Ataxia telangiectasia and Rad3 related
ATM: Ataxia-telangiectasia mutated
BRCA1: Breast cancer type 1 susceptibility protein
BRCA2: Breast cancer type 2 susceptibility protein
CNS: Central Nervous System
DBP: Dibutyl phthalate
MEHP: Mono-(2-ethylhexyl) phthalate
DEHP: Di-(2-ethylhexyl) phthalate
GO: Graphene oxide
PAH: Polycyclic aromatic hydrocarbon
EE2: 17α-ethinylestradiol
BAP: Benzo[a]pyrene
CPT: Camptothecin
